# BK polyomavirus associated progressive multifocal leukoencephalopathy in a person living with HIV

**DOI:** 10.1016/j.bbih.2021.100263

**Published:** 2021-05-05

**Authors:** Brendan O'Kelly, Amy Keane, Emma Devitt, Andrew Lockhart, Deirdre O'Rourke, Fiona Lyons

**Affiliations:** a Genitourinary Medicine and Infectious Diseases Department (GUIDe), St James's Hospital, Dublin 8, Ireland; b Neurology Department St James's Hospital, Dublin 8, Ireland

**Keywords:** Progressive multifocal leukoencephalopathy, BK virus, HIV, Immune reconstitution inflammatory syndrome

## Abstract

Progressive multifocal leukoencephalopathy (PML) is a rare demyelinating disease of the white matter central nervous system occurring in immunocompromised patients particularly those with T cell deficiency such as in HIV, haematological and solid organ malignancies and those taking immunomodulatory medications. PML is caused by JC virus however in rare cases BK virus has been isolated in the cerebral spinal fluid of patients presenting with PML.

In this case we describe a 49 year old man who presented to the emergency department with a 2 week history of progressive right sided weakness and dysarthria. His background history included HIV diagnosed in 2005, he had not engaged with care in the past 2 years and had not been taking anti-retroviral therapy (ART). Other past medical history included untreated hepatitis C. His CD4 count was 90 (11%) cells/mm^3^ on admission and his HIV viral load VL) was 141,000 copies/ml. Magnetic resonance imaging(MRI) showed a hypointense lesion on T1, hyperintense on T2 and FLAIR without diffusion restriction and without mass effect. A lumbar puncture was performed which confirmed JC virus was positive (PCR <50 copies/ml) and also revealed BK virus was positive (PCR 46,511 copies/ml). The patient was commenced on tenofovir alafenamide fumarate/emtricitabine/darunavir/cobicistat in combination with dolutegravir 50mg twice daily. On day 40 post commencement of ART the patient was readmitted with worsening of his right arm weakness and dysarthria. A repeat MRI was performed which showed the hyperdense lesion on T2 and FLAIR appeared slightly larger with some slight enhancement with gadolinium contrast but no other features suggesting PML immune reconstitution inflammatory syndrome (IRIS). The CD4 count had increased to 141(17%) and HIV VL had decreased to 85 copies/ml. A clinical diagnosis of PML IRIS was made and the patient was commenced on prednisolone 30mg BD which lead to an initial improvement in symptoms.

Interestingly in this case, both JC virus and BK virus were detected in the CSF of this patient with the level of JC virus being too low to quantify. BK virus was not detectable on peripheral serum sampling suggesting that BK virus is replicating in the CNS independent of other body sites. There have been 5 case reports in the literature of BK virus as the cause of PML.

Testing for BK virus should be considered in patients presenting with signs and symptoms of PML and encephalitis particularly when no other cause is found.

## 1 Introduction

Progressive multifocal leukoencephalopathy (PML) is a devastating demyelinating disease of primarily white brain matter in immunocompromised patients with impaired cellular immunity, in particular T-cell deficiency (Pavlovic et al., 2018). It is most commonly found in patients with advanced HIV, haematological malignancy, allogenic stem cell transplantation (ASCT), solid organ transplantation (SOT) and in those taking immunomodulatory medications most importantly natalizumab, efalizumab and rituximab (Hung et al., 2020; Major, 2010). Polyomavirus 1 (JC virus), the causative virus of PML, was isolated from brain tissue on the autopsy of the first patient (JC) with the condition and is synonymous with the disease (Padgett et al., 1971). The virus has a predilection for the subcortical U-fibres of the parieto-occipital region but PML lesions can be found in any area of the subcortex and cerebellar peduncles.

Polyomavirus 2 (BK virus) causes tubulointerstitial nephritis, haemorrhagic cystitis, ureteric stenosis, and to a lesser extent pneumonitis and hepatitis in renal transplant patients but also patients post ASCT, SOT, and HIV (Koukoulaki et al., 2009; Reploeg et al., 2001). Both JC and BK viruses are acquired in childhood and are self-limiting respiratory tract infections in immune competent hosts with viral dormancy in kidney, tonsillar, lymphatic and possibly neurological tissues (De Gascun and Carr, 2013). They are double strand DNA viruses of approximately 5 kilobase pairs, have 75% sequence homology and are structurally similar with three domains; 1) an early regulatory domain, 2) a late domain containing VP-1, VP-2 and VP-3 coding capsular proteins, and 3) an additional 400bp non-coding control region (NCCR) (Pinto and Dobson, 2014). The NCCR is conserved in archetypal viral sequences from virus shed in the urine of healthy hosts, and is a binding site for transcription factors including NFκB, necessary for translation of viral proteins. This domain specifically undergoes unique deletion and tandem repeat mutations in immunocompromised patients and generates subpopulations of these viruses in hosts. This is an essential mechanism to confer virulence in these two polyomaviruses; JC virus acquiring neurotropism to cause PML, encephalitis, meningitis and granule cell neuronopathy and BK virus causing renal pathology in patients with impaired cellular immunity; mycophenolate mofetil in particular increases the risk of BK virus nephropathy in the transplant setting (McIlroy et al., 2019; Tan and Koralnik, 2010).

Given the similarities between these viruses it does appear that in some immunocompromised patients BK virus can acquire neurotropic properties and has been found in the CSF and brain tissue of patients with encephalitis, meningitis, meningoencephalitis, retinitis and even PML (Antoniolli et al., 2017; Silva, 2011).

### 1.1 Case

A 48-year-old man presented to the emergency department with a two-week history of progressive right arm weakness, right leg weakness and dysarthria. He described falling off the back of a motorbike as a passenger on acceleration from rest as he was unable to hold onto his companion driver. He had no symptoms of visual/auditory change, no bladder or bowel dysfunction and no swallowing difficulties. The patient felt that he had lost weight but was unable to quantify this. There were no symptoms of fever, chills, shortness of breath, cough, odynophagia, diarrhoea or skin changes to suggest other opportunistic infections.

He provided a comprehensive history and was cachectic. Neurological exam revealed Glasgow Coma Score (GCS) of 15/15, dysarthria without focal cranial nerve deficits, decreased power in his right arm; shoulder abduction grade 4/5, elbow flexion/extension 3/5, wrist flexion/extension 2/5, MTP flexion/extension 1/5, right leg; hip flexion/extend 5/5, knee flexion/extension 4/5, and ankle flexion/extension 4/5. Babinski reflex was positive on the right side, biceps tendon, knee and ankle jerk reflexes were brisk on the right side. Sensation was globally reduced to 10% on the right arm and 30% on the right leg. Neurology was grossly intact on the left upper and lower limbs. No cutaneous, genital or palatal lesions were appreciated. Sub-centimetre nodes in the submandibular region were felt bilaterally.

The patients background history includes HIV which was diagnosed in 2005 on routine screening, CD4 count at time of diagnosis was 551 (17%) cells/mm^3^. He engaged poorly with services due to complex social issues and had been off anti-retroviral therapy (ART) entirely for the previous two years. CD4 nadir was 53 (7%) in April 2020. Other history includes; chronic hepatitis C genotype 1a, pan sensitive non-cavitary pulmonary tuberculosis treated with directly observed therapy in 2011. He was homeless, prior to presentation and actively using intravenous heroin.

**Fig. 1:**
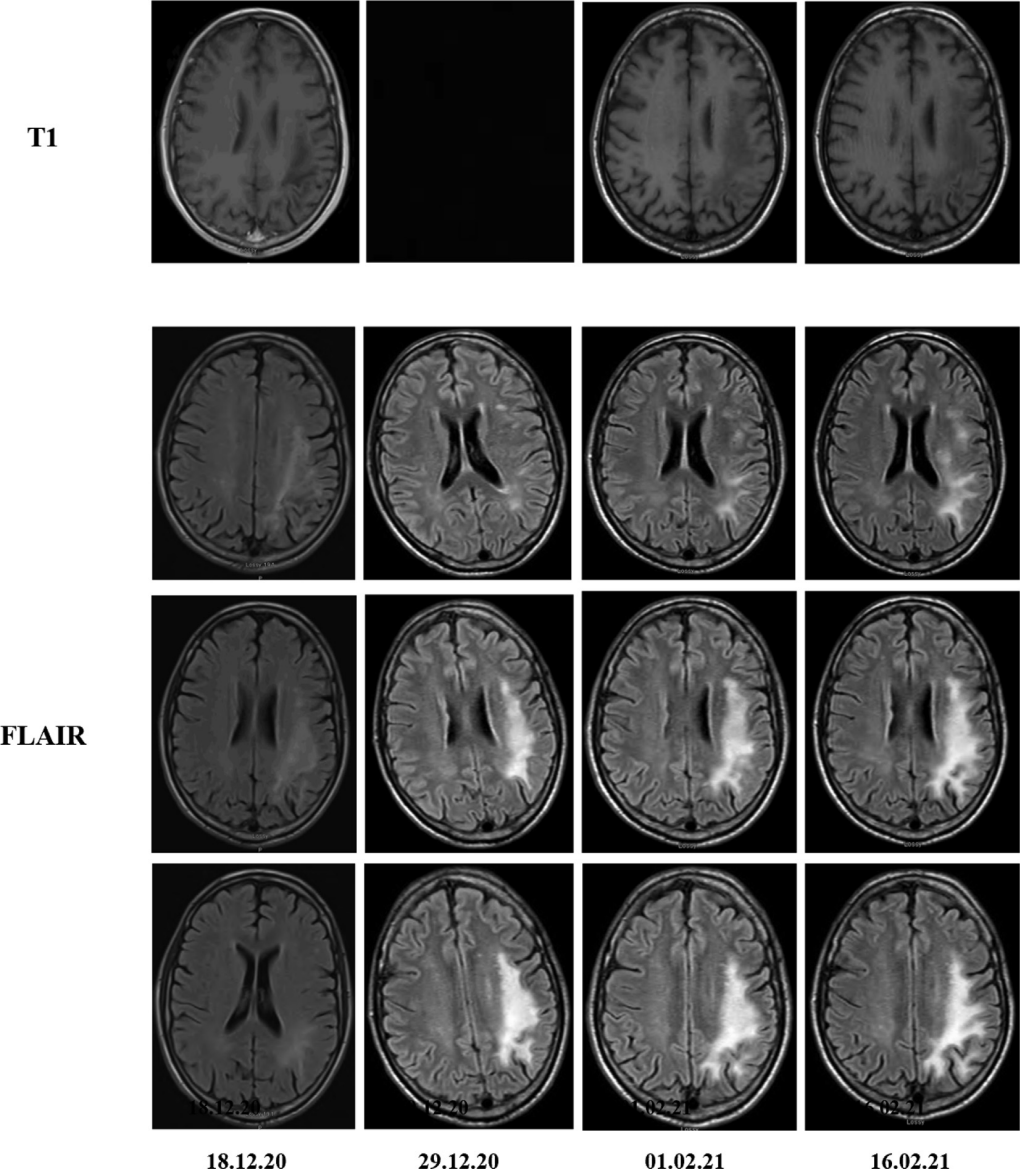
Progression of PML lesion over time on T1 and FLAIR imaging.

A non-contrast computerised tomography (CT) brain scan showed a new ill-defined hypodensity in the left temporo-parietal region compared with a CT from April 2020. Further Magnetic resonance imaging (MRI) showed a hypointense lesion on T1, hyperintense on T2 and Fluid-attenuation inversion recovery (FLAIR) without diffusion restriction and without mass effect (see ​Fig. 1).

Laboratory results on admission were as follows; CD4 90(11%) cells/mm^3^, HIV viral load(VL) 141,000 copies/ml, haemoglobin 8.1g/dL, lymphocyte count 0.6 ​× ​10^9^/L, platelet count 281 ​× ​10^9^/L, EBV IgG positive, serum EBV PCR <800 IU/mL, Syphilis TP assay negative, serum cryptococcal antigen negative, toxoplasma IgG negative. A lumbar puncture was performed which showed a white cell count of 3 ​cells/mm^3^Further cerebral spinal fluid showed protein 36 ​mg/dL, glucose 3.2 ​mmol/L, negative cryptococcal antigen, HIV viral load 1520 copies/ml, JC virus PCR <50 copies/ml, BK virus PCR 46,511 copies/ml and EBV virus PCR 555 copies/ml. A subsequent serum BK virus was negative.

A diagnosis of PML was made and antiretroviral therapy was commenced on day 1 of admission once cryptococcal antigen was found to be negative. Tenofovir alafenamide fumarate/emtricitabine/darunavir/cobicistat once daily combination therapy plus dolutegravir 50mg twice daily was commenced. The most recent genotypic resistance profile was done in 2016, although this showed no resistance mutations this regimen was chosen due to unknown levels of subsequent integrase exposure, the gravity of the current presentation and the necessity to suppress HIV viral replication both in serum and the central nervous system to attempt to improve mortality and reduce further neurological deterioration as quickly as possible.

The patient was monitored as an in-patient and by day 14 post admission no neurological progression or evolving immune reconstitution syndrome were witnessed. A ‘step-down’ bed in an off-site respite facility was arranged in the community with close out-patient clinic follow up. On day 26 post admission the patient was seen in clinic and neurological exam at this time was stable. On day 40 post admission the patient was again seen in out-patients, on this occasion he was noted to have a significant decrease in power in his right arm and leg and worsening dysarthria. Repeat lumbar puncture and routine blood tests were not concerning for a new infection as a cause for this representation. An MRI was done on 01.02.21 which can be seen in Image 1. The lesion was hyperintense on T2 and FLAIR and appeared slightly larger, and although slightly enhancing with gadolinium contrast, no other typical features of PML immune reconstitution inflammatory syndrome (IRIS) were seen. By this time CD4 count had increased to 141 (17%) and HIV VL had decreased to just 85 copies/ml. In this context prednisolone 30 ​mg twice daily was commenced for a clinical diagnosis if PML IRIS, paradoxical worsening of symptoms after commencement of ART. Within one week right arm and leg power and speech had improved; power 3/5 ​at the shoulder, 4/5 ​at the elbow, and 1/5 ​at wrist and MTP joints. Prednisolone was reduced to 20 ​mg once daily but unfortunately the patient deteriorated clinically and repeat MRI brain on 16.02.21 showed extension of the lesion into the corona radiata. Prednisolone was increased again to 40 ​mg twice daily.

## 2 Discussion

In this case we describe a man with advanced HIV who developed classical PML with very high levels of BK virus in his CSF (>40,000 cpm). Interestingly although JC virus was detectable in CSF, this level was too low to quantify (<50 cpm). Furthermore, BK virus was not detectable on peripheral serum sampling suggesting this CSF BKV population is replicating in the CNS independent of other body sites. In this case, BK virus testing was done as part of a full cerebral spinal fluid analysis performed by the National Virus Reference Laboratory.

To date BK virus CNS infection although rare has been described. A review of the literature by da Silva RL et al., in 2011 found 24 cases of BK associated CNS infection eight of whom were self-limiting. Most of these patients had an immunocompromising factor like AIDS, malignancy or transplantation. Of the pathologies found; 9 had encephalitis, 8 meningoencephalitis, 3 meningitis, 2 PML, 2 retinitis. All patients had BK virus positivity on either CSF or brain biopsy or both (CSF n ​= ​20, brain biopsy n ​= ​7). Of those with known outcomes nine passed away. Regarding typical radiological features, BK virus encephalitis has a predilection for periventricular white matter similar to HIV or cytomegalovirus (CMV) and also for pial surfaces and in general was cortex sparing (Silva, 2011). Regarding PLWH, 12 were identified in a review of published cases by Antoniolli et al. including a case described by the authors (Antoniolli et al., 2017). One difficulty that arises with possible cases of BK virus associated neurological pathology is discerning if BK virus is the true cause of a disease process. The presence of BK virus in CSF sampling or even tissue biopsy may be an irrelevant bystander. On closer review of these 12 patients, five had convincing evidence of BK encephalitis, with BK positivity on PCR, immunostaining or southern blot of brain tissues and JC virus negative.

Regarding BK virus as a cause of PML, there are five cases in the literature with variable degrees of evidence to support BK PML (Table 1). All patients are BK PCR positive on CSF and JC virus negative. One 54-year-old man presented with oligo-anuric renal failure and confusion in the context of a renal transplant. MRI findings of bilateral frontal encephalomalacia and gliosis are not entirely in keeping with PML. The immunosuppression was reduced and he improves clinically (Hix et al., 2004). A 29-year-old woman developed symptoms of hemiparesis and hyperreflexia two weeks post-partum. MRI showed lesions that are hyperintense of T2/FLAIR with some GAD enhancement. She failed to improve with steroids and plasma exchange and became obtunded, which is not a typical clinical feature of PML. She improved significantly with adoptive T cell therapy and was discharged (C. Ayvacıoğlu et al., 2019). A 70-year-old man on long term steroids for sarcoidosis developed left sided weakness and homonymous hemianopia. MRI findings of T2/FLAIR hyperintensity in the parieto-occipital region are typical for PML. Steroids were reduced, the overall outcome is not reported (Cabrejo et al., 2005). A 70-year-old woman with Non-Hodgkins Lymphoma, hypogammaglobulinemia and Sjogren's developed 2 weeks of right sided neglect. MRI did show two anomalies consistent with PML. A brain biopsy was performed which was positive for both BK virus and BK antibodies, typical pathological features of PML (bizarre astrocytes and intranuclear inclusions) on staining and JC virus PCR negative. She also died within 1 month of presentation (Daveson et al., 2013). Finally, a 60-year-old lady on methylprednisolone and azathioprine for systemic lupus erythematous presents with confusion, personality change and weakness of lower limbs. T2 hyperintensity was seen with a lesion extending from the left frontal to parietal lobe. Brain biopsy is also BK virus, p53+, cd68+ and JC virus negative. This lady also passed away within 1 month of diagnosis despite reduction of immunosuppression. In 3 of 4 known results, BK virus is also positive in the urine. The latter two cases with BK virus are confirmed on brain biopsy certainly do appear to be irrefutable cases of BK PML.

**Table 1:** Summary of BK PML cases in current literature.

Author	patient	background	Laboratory	radiology	symptoms
JK Hix et al. 2004	54 ​y/o Male	Renal transplantation for diabetic nephropathy	CSF: BK virus +, JC virus – Urine: BK +	MRI: bilateral frontal encephalomalacia and gliosis	Oligoanuric renal failure Aggressive, lethargy, confusion
Cabrejo L et al., 2005	70 ​y/o male	Long term steroids – sarcoidosis	CSF: BK virus +, JC virus – Urine: BK +	MRI: right parieto-occipital. (T1 hypo, T2/Gad enhancement hyperintense)	Progressive lesft sided neurology incl. homonymous hemianopia
Ayvacıoğlu CRK et al. (2019)	29 ​y/o	Post-partum 2 weeks	CSF: BK virus 7000 cpm, JC virus –	MRI: T2/FLAIR positive lesion with some GAD enhancement	Left hemiparesis, hyperreflexia, progressing to loss of consciousness
Daveson KL et al. 2013	71 ​y/o female	B-cell NHL, Sjogren's, hypogammaglobulinemia	CSF: BK virus 11,975 cpm, JC virus – Urine: BK + Brain bx: BK PCR +, JC – BK Ab +	MRI: two areas of abnormality in the posterior left frontal lobe in the subcortical and the periventricular regions	Ataxia, 2 weeks of right sided neglect
Melis M et al. 2018	60 ​y/o female	SLE, azathioprine and Medrol	CSF: BK virus 1.3 ​× ​10^8^cpm JC virus PCR – BK blood 6.3 ​× ​10^5^ cpm BK urine -neg Brain bx: p53+, Cd68+, JCV-, BK virus 1.1 ​× ​10^8^	T2-hyperintensity in the left frontal lobe with extension on the right frontal and parietal lobes with periventricular extension and on the corpus callosum genu and DWI-restriction	Confusion, personality change, weakness of lower limbs bilaterally

In summary the authors would like to highlight the neurotropic properties of BK virus in immunocompromised hosts and to consider testing for this virus in the context of negative results for other known causative viruses of encephalitis and PML; JC; CMV; HIV and HSV. At present immune reconstitution of the host and mitigation of immune mediated inflammatory syndromes are at the forefront of treatment and whether PML is caused by JC or BK may be academic as our current therapeutic toolkit is not sufficiently advanced for directed therapy. Small studies have shown that potentiating the immune system with immune checkpoint inhibitors like pembrolizumab show promise for PML (Cortese et al., 2019). Similarly, immune ‘boosting’ with BK or JC virus specific T-cells from healthy HLA matched donors have also shown promise and directed therapy with the respective specific T-cell populations may be where the difference lies for future therapy and the importance in discerning BK for JC virus associated PML (Muftuoglu et al., 2018; Steinhardt et al., 2020).

## Funding

No funding was received for the preparation of this manuscript.

## Declaration of competing interest

The authors have no conflicts of interest to declare.
